# Advances in Nanofabrication Technology for Nutraceuticals: New Insights and Future Trends

**DOI:** 10.3390/bioengineering9090478

**Published:** 2022-09-16

**Authors:** Rachitha Puttasiddaiah, Rohitha Lakshminarayana, Nandini Lalithadripura Somashekar, Vijai Kumar Gupta, Baskaran Stephen Inbaraj, Zeba Usmani, Vinay Basavegowda Raghavendra, Kandi Sridhar, Minaxi Sharma

**Affiliations:** 1 P.G. Department of Biotechnology, Teresian College, Siddarthanagar, Mysuru 570011, India; 2 Center for Safe and Improved Food & Biorefining and Advanced Materials Research Center, Scotland’s Rural College (SRUC), Kings Buildings, West Mains Road, Edinburgh EH9 3JG, UK; 3 Department of Food Science, Fu Jen Catholic University, New Taipei City 242 05, Taiwan or; 4 Department of Applied Biology, University of Science and Technology, Meghalaya 793101, India; 5 UMR1253, Science et Technologie du Lait et de l’œuf, INRAE, L’Institut Agro Rennes-Angers, 65 Rue de Saint Brieuc, F-35042 Rennes, France; 6 Laboratoire de Chimie Verte et Produits Biobasés, Département Agro Bioscience et Chimie, Haute Ecole Provinciale de Hainaut-Condorcet, 11, Rue de la Sucrerie, 7800 Ath, Belgium

**Keywords:** nano-formulation, nutraceuticals, prebiotics, nanofabricated delivery system, liposomes, nano-emulsions

## Abstract

Bioactive components such as polyphenolics, flavonoids, bioactive peptides, pigments, and essential fatty acids were known to ward off some deadliest diseases. Nutraceuticals are those beneficial compounds that may be food or part of food that has come up with medical or health benefits. Nanoencapsulation and nanofabricated delivery systems are an imminent approach in the field of food sciences. The sustainable fabrication of nutraceuticals and biocompatible active components indisputably enhances the food grade and promotes good health. Nanofabricated delivery systems include carbohydrates-based, lipids (solid and liquid), and proteins-based delivery systems. Solid nano-delivery systems include lipid nanoparticles. Liquid nano-delivery systems include nanoliposomes and nanoemulsions. Physicochemical properties of nanoparticles such as size, charge, hydrophobicity, and targeting molecules affect the absorption, distribution, metabolism, and excretion of nano delivery systems. Advance research in toxicity studies is necessary to ensure the safety of the nanofabricated delivery systems, as the safety of nano delivery systems for use in food applications is unknown. Therefore, improved nanotechnology could play a pivotal role in developing functional foods, a contemporary concept assuring the consumers to provide programmed, high-priced, and high-quality research toward nanofabricated delivery systems.

## 1. Introduction

According to the description placed by National Nanotechnology Initiatives (NNI) in 1999, nanotechnology is defined as the appliance of systematic scientific knowledge to operate and manage matter to the nanoscale range to draw on size and structure-dependent properties and phenomena [1]. Food nanotechnology is an upcoming, promising, and fast-growing field bound to an extensive range of disciplines and has established an assortment of applications in the field of food sectors. Food nanotechnology has come into play for the last 10 years and globally opened up new opportunities and promises for novel discoveries in the Food industry. Numerous bioactive components from the Foods are well known from the ancient time for their beneficial activity for maintaining a healthy lifestyle, management of diseases, and also for their in-vitro biological functionalities; in contrast to this, many of the bioactive ingredients are not inevitably active after being ingested or consumed as part of food ingredients. The modern medicine system includes expensive disease treatment methods. People are unsatisfied with moving toward alternative substitutes (beneficial products or prebiotics and probiotics) in maintaining a healthy diet to protect against diseases [2]. Nutraceuticals are beneficial compounds generally attained from nutrients, herbal products, dietary supplements, and diets for genetically engineered “designed foods”. Food products include soups, cereals, and beverages [3,4].

Physico-chemical stability of these bioactive compounds and nutraceuticals are significantly affected by the harsh environmental condition during production, processing, and storage. In the meantime, due to the physiological conditions generated within the body system during digestion and absorption, the organic functions of bioactive compounds are altered. With an ever-rising demand for healthy foodstuff, substantial interest has been drawn to renovating food processing technologies to build up novel, purposeful, or functional food products with enhanced health benefits. In this perception, nanotechnology or nanofabricated bioactive compounds/nutraceuticals with superior delivery strategies have been launched in the field of the food sector to improve the biological efficacy and physicochemical stability of naturally available bioactive compounds/Nutraceuticals in food and also to shield the food quality [1,3]. Nanofabrication is a promising and exceptional tool in food biotechnology to augment the efficiency of biomolecules in the past, present, and future.

**Table 1:** Biochemical profiling of principle bioactive compounds and their functions.

Bioactive Compounds	Health-Promoting Property	Occurrence	Molecular Weight (g/mol)	Structure	Reference
Quercetin	Promoting cardiovascular health properties and helping in blood flow.	Fruits and vegetables, especially in onions, grapes, lemon tea, citrus, etc.,	302.236	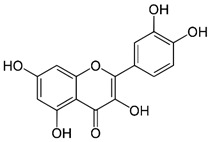	[5]
Luteolin	Anticarcinogenic activity	Green pepper carrots, Broccoli, oregano	286.24	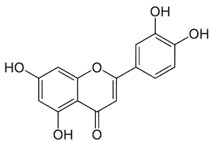	[6]
Kaempferol	Antioxidant activity and Anticarcinogenic activity	Tomatoes, apples, grapes, green tea, broccoli, lettuce, peaches	286.23	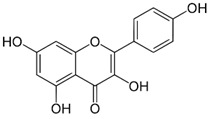	[7]
Curcumin	Antibacterial activity, antioxidant activity, and anti-inflammatory activity	Turmeric	368.38	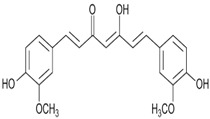	[8]
Berberine	Treatment of breast cancer, colon cancer, pancreatic cancer, gastric cancer, liver cancer, oral cancer, etc.,	Widely present in barks, leaves, twigs, rhizomes, roots, and stems of several medicinal plant species.	336.3612 g/mol	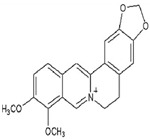	[9]
Rutin& Quercetin	Protection against cancer and some other diseases. Lowers cholesterol, mainly used in skin aging.	invasive plant species, *Carpobrotus edulis*	610.517 g/mol	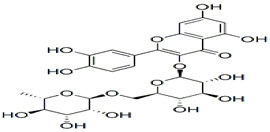	[10]
Astaxanthin	Protection from UV skin damage, Reduction in inflammation, Supports Immune system.	algae, yeast, salmon, trout, krill, shrimp, and crayfish	596.841 g/mol	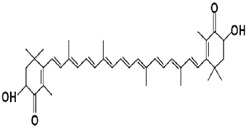	[11]
Vitamin E	helps maintain healthy skin and eyes, and strengthen the body’s natural defense against illness and infection (the immune system).	Plant-based oils, nuts, seeds, fruits, and vegetables.	430.71 g/mol	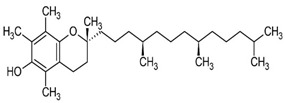	[12]

## 2. Nano Formulation of Bioactive Compounds

**Figure 1:**
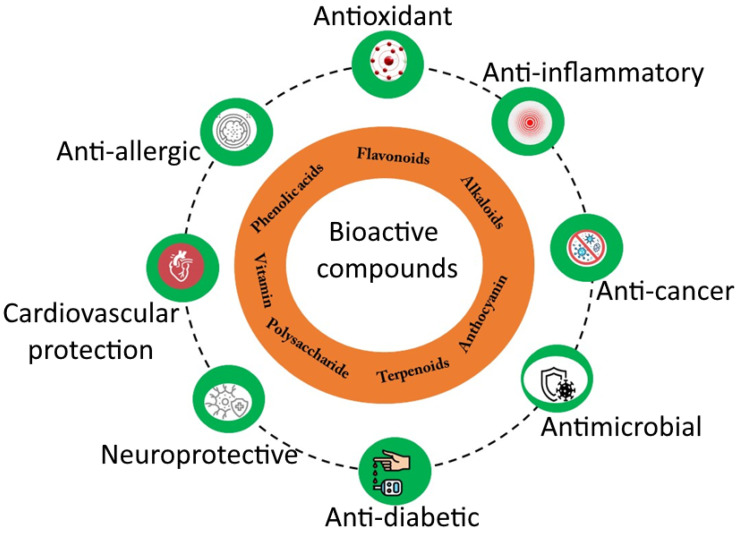
Bioactive compounds for different applications.

Bioactive compounds such as vitamins, flavonoids, phenolics, proteins, etc., are essential for proper cell growth and differentiation in living organisms; these also play a crucial role in combating diseases by scheming biological mechanisms (Figure 1 and Table 1). Recent studies on coupling the bioactive compounds with existing drugs such as cyclophosphamide and paclitaxel have enhanced breast cancer’s healing effect [13]. Consequently, combining medications with bioactive compounds opens a new window in the medical field to treat various diseases. In essence, the formulation of bioactive molecules and drugs also complements the negative aspect of the overdose of drug molecules [14]. On the other hand, the desired amount of bioactive compounds is not consumed in some cases. As a result, unregulated uptake also leads to deterioration in health. Henceforth functional food or food supplements are formulated to tackle these issues. Out of this biological importance, other fields such as biomedical, optoelectronics, and health care with light modification and functionalization were also the beneficiaries. They offered an excellent demand for bioactive compounds. Hence the development of nano-formulation bioactive compounds with an improved adaptation of nanofabrication tools of delivery strategies is in great need or challenge in scientific research [14,15]. Advancement in nano-formulation procedures certainly reduces the limitation of bioactive molecules such as bioavailability, specificity, and solubility. That paves new insights into discoveries of novel materials principally apt for diverse biomedical applications (Table 2).

### 2.1. Nutraceuticals and Nanobiotechnology

Nutraceuticals are those beneficial compounds that may be a kind of food or part of food that has come up with medical or health benefits. Prevention and treatment of diseases are also involved in these nutraceuticals [2,26]. Moreover, consumers are more conscious of their health care and its administration and management. In approach to modern medicine, people are discontented with expensive disease treatment, so people are often moved towards substitutes of beneficial products. This makes nutraceuticals predominantly appealing. Nutraceuticals are beneficial compounds generally attained from dietary supplements, nutrients, herbal products, and diets for genetically engineered “designs foods”. Also, food products such as soups, cereals, and beverages [4,27]. In the current years, researchers have bought with a new plan that put forth their effort in bringing up an improved diet by substituting modern medicine with natural products. Furthermore, the R & D related and nutraceuticals market is vastly evolving [28,29].

Nutraceuticals have been widely used in nanotechnology due to their ability to improve bioavailability, ingredient solubility, and stability. There are various unique properties associated with nanoparticles that involve small size and a high surface-to-volume ratio. Nanomaterials possess physio-chemical properties that show unfavorable effects on human health [30]. There are specific safety measures to bring up varieties of new marketable nutraceuticals, and formulation estimation of their efficacy is required. In this article, in agreement with European food safety Authority recommendations and high throughput screening fashion, they have worked upon an in-vitro-based approach that evaluates the safety and efficacy of new formulations.

This type of study plays a significant role in its time and cost-effectiveness. It is an essential tool that helps support companies as it comes up with the design of a safe concept presently under development in Europe [31]. In the field of nanoscience, food industries and nutraceuticals open a wide variety of technology to this. There has been explained with the new probability by nanotechnology in consultation to modified properties of nutraceuticals and its application in the development of nutritional supplements, food industry, and nano-formulations. In nanotechnology, nanoparticles have got various properties that complete the application in the food sector and nutraceuticals [32].

This review article is required to present advanced nano-based nutraceuticals with enhanced solubility, bioavailability, efficiency, improved encapsulation, sustained and targeted drug, increased stability, microbial contamination with improved pharmacological activity, and protection against degradation [33]. This article is also helpful in the knowledge of how nanomaterials for encapsulating enzymes, antibodies, peptides, etc., and nanoparticles play an essential role in the application of the food packaging industry. There are enormous benefits that have come across with nanotechnology and nanomaterials in health sectors by improving quality of life and treatment strategies [34].

### 2.2. Definition and General Classification of Nutraceuticals

In 1989, Stephen De Felice M.D derived the term nutraceuticals from ‘nutrition’ and ‘pharmaceutics’ [35]. A nutraceutical is defined as any substance that is a food or a part of food and provides health or medical benefits, including the treatment and prevention of disease. The term Nutraceutical can be used as a marketing term. Dietary supplements such as MSM, Glucosamine, and chondroitin are most popular and are used for joint health. Functional food for one consumer can act as a nutraceutical for one another. Examples of nutraceuticals include fortified dairy products (e.g., milk) and the city’s fruits (e.g., orange juice). Several nutraceuticals are studied under cancer therapies and are beneficial in curing those diseases [36,37]. Nutraceuticals’ definition mainly depends on their source. They are broadly classified primarily based on their pharmacological condition, the chemical constitution of the products, and natural source. Nutraceuticals are commonly classified into dietary supplements, medicinal foods, pharmaceuticals, and functional foods [38,39].

### 2.3. Dietary Supplements

Dietary supplements are the type of nutraceuticals that contain nutrients derived from food products. These dietary supplements are intended to complement or supplement the diet and are distinct from traditional foods [40,41]. Dietary supplements include capsules, pills, tablets, powders, liquids, or extracts. It contains many valuable supplements that include minerals, vitamins, herbs, amino acids, fiber, enzymes, and other plants. These dietary supplements are regulated or controlled as foods by the U.S. Food and Drug Administration [FDA]. Because of their critical role in cellular growth and biotransformation or metabolism, membrane shape, and function, LCPUFAs are extremely important for prenatal and infant growth and development [42]. Given that they are high in arachidonic acid and docosahexaenoic acid, the LCPUFAs (“walnuts, flaxseed oil, soybean oil and canola oil”) help in promoting brain growth and irreversible damage [43]. The five essential fatty acids for human consumption are saturated palmitic acid, stearic acid, monounsaturated oleic acid, polyunsaturated linoleic acid, and linolenic acid [44]. Arachidonic acid, eicosatetraenoic acid, and docosahexaenoic acid are newly synthesized secure, less exorbitant, and feasible plant-based products of these fatty acids [45]. Higher concentrations of eicosatetraenoic acid and docosahexaenoic acid were generated in *Brassica juncea* through the use of an extreme acquiesce [43]. These dietary supplements also include medicines and other products [46].

### 2.4. Functional Foods

Functional foods offer health benefits beyond their nutritional value, including whole foods and fortified [47,48]. Proponents of functional foods also enriched with dietary components say that they promote optimal health and may help reduce the risk of chronic diseases. It gives health benefits compared to its traditional nutrients [49]. According to the Canadian Health Organization (CHO), functional foods are “regular recipes that seem to have elements introduced in order to give it a proper scientific or physiological advantage, aside from a merely dietary effect”. According to Japan, all pragmatic ingredients have to be available and arising in their herbal version [43] instead of a tablet, capsule, or powder [50] consumed in the nutrition plan on even a regular or daily basis [51] and should revise a biological system in order to avoid disease or disorder-like instances [43].

### 2.5. Medicinal Nutraceuticals and Pharmaceuticals

Medicinal foods are specially formulated and intended for the dietary management of a disease that has acquired distinctive nutritional needs and cannot be met by the regular diet itself [52]. These medicinal foods are absorbed internally under the consultation of a qualified physician. It acts as precise dietary management of a particular disease under which the nutritional requirements of individuals are confirmed by medical evaluation and based on recognized scientific principles [53]. Pharmaceuticals are used in diagnosing, preventing, or treating the disease and for modifying, restoring, and correcting organic functions [54]. These are enormously helpful medical products obtained from animals or crops. The term is derived from the combination of the words ‘farm’ and ‘pharmaceuticals’ [55]. Consumers’ well-being is increasing, with a focus on scientific research and asserts reassurance of bioactive molecules, along with novel therapeutic dose and distribution forms [56,57]. The ability of innovative medical and drug technologies to diagnose disease and eventually expand the average lifespan has also provided us with a unique outlook. All food products could have nutritious, sensory characteristics, and preventative measures features. Countless biologically active components have already been commercialized as pharmaceuticals (pills, capsules, solutions, gels, liquors, powders, granules, etc.). These are known as “nutraceuticals”, and they help to improve public health. These provide a physiological reward or grant immunity against serious illness, support healthy, and slow the aging process [56,57].

### 2.6. Role of Nutraceuticals in Human Health

Nutraceuticals are concentrated compounds from food sources that can provide health benefits, including disease prevention and health promotion [58]. Examples of nutraceuticals include substances such as antioxidants, probiotics, and omega 3. It combines nutrition and pharmaceuticals [59]. Nutraceuticals play a preeminent, significant, and valuable role in maintaining normal physiological function, which helps to keep healthy human beings; nutraceuticals are part of food [60]. There has been a fast growth in the nutraceuticals market worldwide that are present in the population and the health trends. In nutraceuticals, the food products used can be classified as fiber, polyunsaturated fatty acids, and other diverse types of herbal/natural foods [61]. Nutraceuticals are the source that is crucially utilized to regulate some of the century’s major problems that involve cancer, diabetes, cardiovascular diseases, osteoporosis, cholesterol, arthritis, etc. [62,63]. Furthermore, nutraceuticals have led to a new era of health and medicine in the food industry that has become a research-oriented sector. In this decade, people are more conscious of the correlation between life-threatening diseases and eating habits differing and increasing daily [29]. For this reason, people have become more attentive to the quality consumed and show interest in the foods that have beneficial effects on one’s health and avoid the onset of life-threatening diseases [64,65]. The above features help the researchers study the potential effects of beneficial nutraceuticals on their mechanism of action and human health. On an identical note, industries, mainly the food industry, were much more prominent and were interested in developing food products that attract consumers or people to purchase them for their health [66,67].

It is a comprehensive technology that has allowed researchers to improve enormous limitations related to the use of nutraceuticals to retinue their encapsulation into these structures, which involves their stability, poor bioavailability, and low solubility [68,69]. It is mandatory to know about the fact of bioavailability on a notice that it’s a fraction of taken compound, which is absorbed and available for physiological functions and is the critical characteristic for nutraceuticals compounds, because of its effectiveness which is strictly associated to their bioavailability [70,71]. Few other important factors deal with the bioavailability of nutraceuticals, and the factors are various endogenous and exogenous, which are involved in the biochemical transformation that undergoes into food storage, Epithelial cells, physio-chemical features, and so on [72,73,74]. Due to this, more advanced technologies have been established to exert their beneficial effects when introduced into the organism. Different nano-formulations have been developed between them to increase the needful effects of nutraceuticals [75,76]. In the presence of the highest quantity of bioavailability, there promotes a controlled release and targeted delivery of encapsulated bioactive compounds, which increases the biological efficacy of food. Nanotechnology develops the framework of the agriculture and food industry by offering various advantages [77,78]. To design the nutraceutical delivery system, multiple criteria should be enrolled into an account which involves the formulation it must have good physical and chemical properties, sustainable production costs, and food-grade materials used [79,80].

### 2.7. Favorable Nutraceuticals Properties on Human Health

Nutraceuticals are those substances that have a physiological benefit or protect against chronic diseases. Most therapeutics are involved in it and have had various beneficial effects on human health in recent years. These includes diabetes, arthritis, inflammation, cholesterol, and many other disease conditions [81,82]. One of the best nutraceutical products is natural honey which can be used to feed infants, as it is known to be a food with great benefits and beneficial nutritional properties for human health [83]. Natural honey is made up of antioxidants and enzymes that are essential to the digestive process. Using raw honey allows for cloudiness. Since the extract inhibits the angiotensin-I converting enzyme (ACE1), it is also used to treat high blood pressure. Finally, it is important to emphasize that the skin of the allis shad, which is a nutraceutical, has a reduced benefit compared to some traditional medicines that have been used in the treatment of type 2 diabetes as conditions such as cardiovascular disease and eye disease Side effects on the patient provides problems and ulcers [84]. The honey obtained has antimicrobial activity and can be used to disinfect the infected wound [85]. Thanks to the increase in experimental evidence of the efficacy of nutraceuticals in the prevention or treatment of various pathological conditions, numerous efforts have been made to apply nanotechnology to encapsulate these natural products [86]. A combination of nutraceuticals (bergamot extract, chlorogenic acid, phytosterols, and vitamin C) was administered to overweight subjects with dyslipidemia, increased glucose, and lipid metabolism compared to subjects treated with overnight placebo [87]. Numerous scientific studies have conclusively shown that controlling blood cholesterol levels can reduce the likelihood of developing cardiovascular diseases [88,89,90]. It is unacceptable that the food industry is responsible for producing a significant part of its waste. It has a major impact on the environment and can be used as an essential source of nutraceuticals. The example concerns a citrus peel, fruit waste rich in phenolic compounds with strong antioxidant activity [87,91]. The antioxidant activity of natural compounds has multiple beneficial effects on human health [92].

Antioxidants act as a barrier to protect the organism and as free radical scavengers, preventing oxidative damage [93,94]. In addition, their effectiveness helps prevent cancer and cardiovascular disease, and their anti-inflammatory effects and ability to establish and prevent Alzheimer’s disease are better known [95]. It has been scientifically proven that phenolic compounds isolated from the lily of the valley (*Citrus maxima*) benefit citrus peel. Honey, for example, has a significant antibacterial effect and can be used to disinfect infected wounds. It also shows inhibitory activity against amylase and glucosidase, indicating its usefulness in treating type 2 diabetes [96]. The extract is also used to treat high blood pressure as it inhibits angiotensin I converting enzyme (ACE1) [97]. Finally, because the shells of the Maddocks are marked. Since the extract inhibits the angiotensin-I converting enzyme (ACE1), it is also used to treat high blood pressure. Finally, it is important to emphasize that as a nutraceutical, the shell of the allis shad offers the benefit of reducing side effects for the patient; compared to some traditional drugs that have been used as nutraceuticals in the treatment of type 2 diabetes, it has the advantage of having fewer side effects on patients than other typical drugs used to treat type 2 diabetes [98]. Another study by Barreca et al. [99] showed how antioxidants can be extracted from pistachio waste; Only by using organic solvents was it possible to extract about 20 compounds with cytoprotective and antioxidant properties from the ripe pistachio shell [100]. They are catechin, gallic acid, isoehamnetin-3-O-glucoside, quercetin-3-O-rutinoside and naringin [101]. In addition, nectarines, olive leaves, and tomato skins were considered, since the new claims of nutraceuticals and additives [102] have currently been detected in the polyphenolic extracts from these food wastes, which confirmed a specific positive activity in insulinemia and postprandial glycemia [103].

## 3. Nanotechnology as a Nutraceutical Properties Enhancement Strategy

Credit to the increase of experimental data on the efficacy of nutraceuticals in preventing or treating several pathological states [97], a great deal of effort has gone into applying nanotechnology to encapsulate such natural products [104]. The encapsulation era is a very promising method in nutraceutical delivery because it offers several advantages [105]. A unique interest has been drawn internationally to *Eucalyptus* plants in various fields of industry. Amongst them are prescription drugs, perfumery, nutraceuticals, and fixtures. Consequently, they represent a quick-developing supply of wood and a source of oil used for several [106].

**Figure 2:**
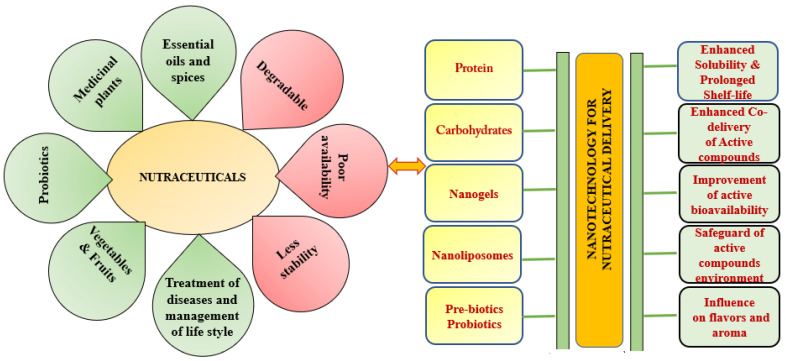
Applications of nutraceuticals and nanotechnology for nutraceuticals delivery.

Nanoencapsulation techniques produce active component nanosuspensions with a coating or encapsulated with either wall materials in dried or liquid [107] The chemical structure of nutraceuticals can be safeguarded with the possibility of associated environmental agents, which involves light, oxygen, pH, radicals, and temperature. It enables site-specific delivery, increases bioavailability, and permits the controlled release of encapsulated compounds (Figure 2) [108]. Regarding the ability of nanosystems to withstand the release of supplied active compounds, it is bound to the beneficial site of action and encapsulation of nutraceuticals with a specific time or concentration release profile, and it is the main challenge that has to be performed [109].

Subsequently, an ideal delivery system must exist and be able to release its contents, leading to certain stimuli that involve enzymes, moisture, pH, temperature, and preserving nutraceuticals from the same stimuli [55]. In addition, the encapsulation of nutraceutical compounds leads to an enrichment of their solubility; as a result, when nutraceuticals are loaded into the carrier, the characteristics obtained depend in particular on the physicochemical properties of the vesicle relative to an entrapped compound [96]. Nanosystems also offer the possibility of adding lipid and water-soluble molecules, thus supporting their synergistic effect [110]. They also show promise in physiochemical stability and help avoid undesirable taste and odor changes that can result from adding nutraceuticals to food products. In a drug delivery system, the materials used to implement it can be of a variety of natures (lipid, polymeric, protein), so long as they are generally recognized as safe status (GRAS) [111]. In addition, the mode of drug delivery and its composition are preferred based on the chemical and physical properties of the encapsulated compound, the target to be reached, and its application type [112]. Accordingly, nanoencapsulation of nutraceuticals has been shown to have beneficial adverse effects on human health, thus helping to reduce side effects [96]. Main nanocarriers, mainly used for encapsulating nutraceuticals, have to be analyzed.

### 3.1. Nano Probiotics

Micro and nano probiotics are live micro-organisms intended to have health benefits when consumed or applied to the body. Examples of probiotics involve yogurt, kefir, sauerkraut, tempeh, kimchi, etc., probiotics have become a field of interest to research in recent times [113]. It is considered a desirable alternative to antibiotics because of their beneficial effects on the economy and the safety of farm animals [114]. The International Scientific Association for Probiotics and Prebiotics (ISAPP) has introduced a wide range of products containing probiotics that have to contain health-promoting effects. It includes drugs, baby formulas (e.g., first milk), therapeutic supplements, and foods (e.g., fermented dairy with reportedly health-beneficial effects) [115]. Probiotics come from different sources that involve several foods, natural environments, and human guts. The main property of probiotics is the ability to survive through the gastrointestinal tract; it promotes health benefits to the host etc., [116] (Table 3). The probiotics can also include fungal and yeast species such as *Saccharomyces cerevisiae* and *Kluyveromyces*.

### 3.2. Nano Prebiotics

Nano-prebiotics are foods that promote the growth of beneficial bacteria in the gut. These are a type of fire that the human body cannot digest; they serve as food for probiotics and are tiny living organisms that involve bacteria and yeast (Table 4).

Prebiotics are found in various sources, including non-digestible carbohydrates, non-digestible oligosaccharides, unrefined wheat, unrefined barley, and soybeans [125]. Some compounds found in prebiotics are xylooligosaccharides, lactulose, and non-starch polysaccharides (NSP) [126].

### 3.3. Food Grade-Nanofabricated Delivery Systems

Incorporating functional food components for the better functionality of food products to meet consumer needs for a healthy lifestyle is in great demand. It has become a delightful trend in the market. These programmed food or functional food components is not limited only to their inhibitory action against unsolicited microbial growth. Still, it enhances the product’s taste, flavor, and color and attributes beneficial health effects. Active food constituents possess some properties such as low water solubility, unappetizing sensory character, constrained oral bioavailability, and intolerance to chemical degradation, so most of these functional food constituents are antagonistic to the food matrix. Nanoencapsulation technology deals with encapsulating bioactive compounds using different nano-fabricated delivery systems [127]. Even though these delivery systems can be used as biocompatible and biodegradable to make them reachable for food applications, these delivery systems are generally classified into carbohydrates, lipids, or protein-based [128].

### 3.4. Carbohydrate/Polysaccharides Delivery Systems

**Figure 3:**
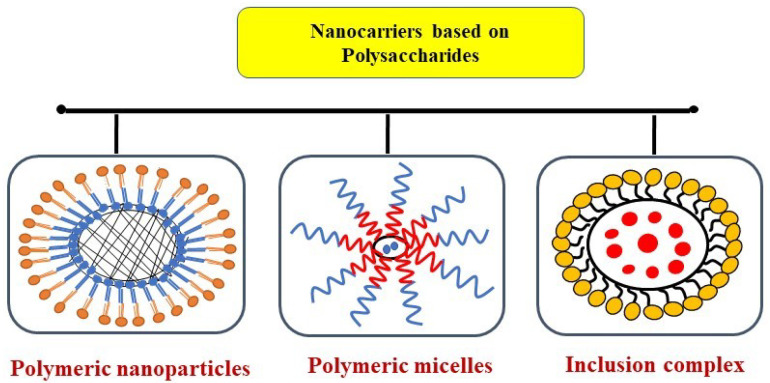
Polysaccharides-based nanofabrication systems.

Carbohydrate delivery systems can interact with a wide range of bioactive components because these carbohydrate-based delivery systems possess exceptional properties like biodegradability, abundance, and a high potential for adaptation to enviable functionality so that these delivery systems are more appropriate for high-temperature stability over lipid and protein-based carriers which undergo degradation and denaturation. Cyclodextrin (α, β, γ) is a carbohydrate-based delivery system. It is a prominent condensed cone-shaped oligosaccharide with a hydrophobic central cavity and hydrophilic end on the other. So, in carbohydrate-based delivery systems, the present trend is to study these cyclodextrins. These are useful for the confinement of less soluble, temperature susceptible, or chemically liable food bioactive. On the other hand, a mixture or combination of two or more carbohydrate-based delivery systems gives inventive frontiers for increased efficiency. Nanofabrication techniques like spray drying, electro-spraying, coacervation, and electro-spinning are extensively used to fabricate carbohydrate-based delivery systems [129,130] (Table 5) (Figure 3).

### 3.5. Protein-Based Delivery Systems

Protein-based delivery systems also have captivated great recognition. The exterior surface of protein-based delivery systems possesses many functional groups that facilitate their ability to encapsulate both hydrophilic and lipophilic bioactive constituents. They are mainly obtained from plant, animal, and microbial sources. Coacervation, gelation, spray drying, and electro-hydrodynamic process are commonly used methods for fabricating protein-based delivery systems. [135]. In recent years plant-protein-based delivery systems have also emerged in the field of nanotechnology to shield and manage lipophilic bioactive compounds (polyphenols, polyunsaturated fatty acids, vitamins, carotenoids), has increased attention in nutraceuticals, food, and pharmaceutical fields.

**Figure 4:**
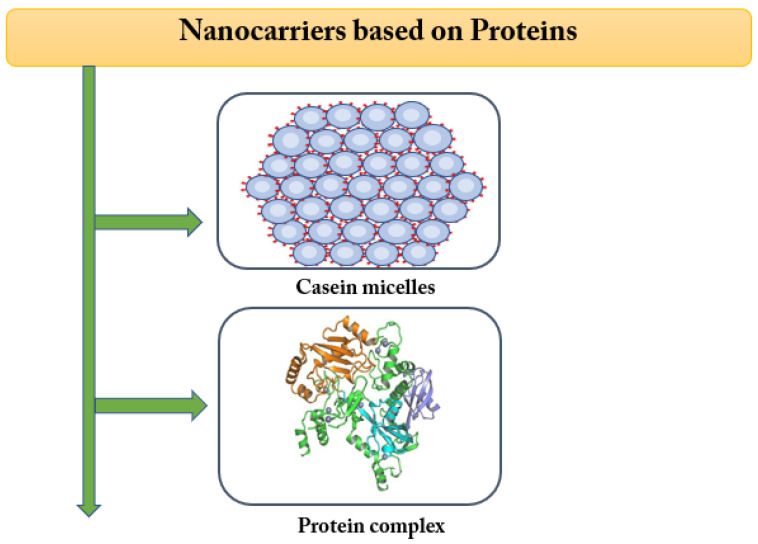
Protein-based nanofabrication system. Adapted with permission from Ref. Singh et al. [136]. Copyright © 2021, Controlled Release Society).

The promising role of plant proteins from legumes, nuts, tuber, oil/edible seeds, and cereals is aggravated by their sustainable, eco-friendly, and vigorous nature collating with other sources. Before using raw materials carriers, particular challenges need to be addressed to overcome the adverse consequences by modifying approaches to improve their techno-functionality and limitations, aiming to fabricate innovative production of plant-based carriers (emulsions, hydrogels, structures, and self-assembled films). This plant protein delivery system is a limited application that only acts as a carrier for lipophilic compounds. Nevertheless, bottomless insights into new purification or extraction technologies and protein sources are necessary to validate their functional properties for the evolution of an effective delivery platform [136] (Figure 4).

The spatial shape, size, colloidal stability, water dispersibility, etc., of protein-based nanocarriers, were determined by the physicochemical molecular principles of protein. Some of the available preparative methods could be used to fabricate several protein-based nanocarriers, including antisolvent precipitation, pH–driven, gelation, and electrospray methods. Recent studies have evidenced that protein-based nanocarriers provide numerous advantages in aid of the application of bioactive compounds to the food, medical, and cosmetic sectors [137]. 

### 3.6. Lipid-Based Nano Delivery Systems

**Figure 5:**
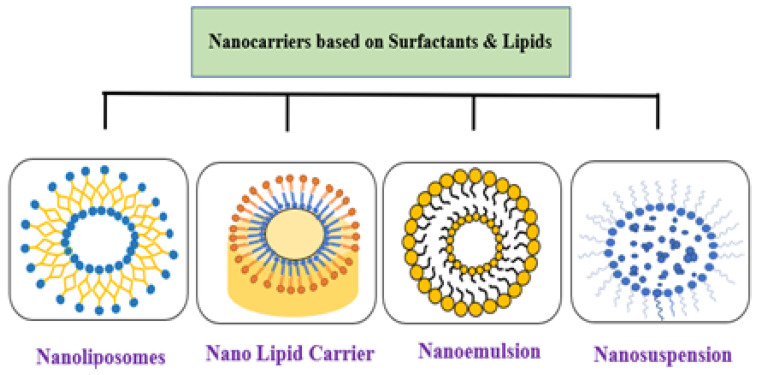
Lipid-based nanofabrication system.

Compared to protein and carbohydrate-based delivery systems, the lipid-based nano delivery system is the most auspicious encapsulation system that offers peerless advantages over the other two systems due to its ability to entrap materials with different solubilities enhanced targeted delivery and ability to guard a constituent against free radical’s pH and enzymes [138]. Lipid-based nanocarriers have been fabricated for food applications such as nanoemulsions, nanoliposomes, nanostructured lipid carriers (NLC), and solid lipid nanoparticles [139] (Figure 5).

### 3.7. Nano-Emulsions

Nano-emulsions are liquid droplets with colloidal dispersion sizes less than 100 nm. They can be either oil-in-water or water-in-oil (w/o) [140]. They are made by entrapping bioactive constituents within the distribute phase, which is self-stabilized by surfactants/biopolymers in the continuous phase [141]. They are made by entrapping bioactive constituents within the distribute phase, which is self-stabilized by surfactants/biopolymers in the continuous phase [141]. These can be manufactured using high-energy emulsification techniques such as homogenization, ultrasonication, and microfluidization.

### 3.8. Nanoliposomes

Liposomes are nano-sized spherical vesicles composed of a phospholipid, a lipid bilayer with an aqueous core. Nanoliposomes have a superior surface area and tolerable stability profile to safeguard their size within nanometric scales such as small liposomes (20–100 nm) and large liposomes (>100 nm) [105]. These carries mainly contain lipids and phospholipids. Conversely, some include molecules such as antioxidants, carbohydrates, protein, or sterols in their structure. Their amphiphilic nature makes them potent nanocarriers since these have the potential to encapsulate and massive release hydrophobic and hydrophilic compounds concurrently, providing mutual benefit. Distinguishing phospholipids/bilayer structure is highly attuned to the skin surface, allowing them to act as penetration enhancers of nutraceuticals and bioactive compounds towards targeted sites [142]. Some techniques such as microfluidization, extrusion, and ultrasonication are usually used for nanoliposomes production [143]. The main restrictions lie with poor solubility towards low pH and enzymes after intake, low loading ability, and higher cost of food-grade fabrication wall materials. The present trend is to tackle these barriers for the expansion of nanoliposomes optimistic nanocarriers on a large scale [144].

Examples of drugs in liposomal carriers are doxorubicin, amphotericin B, nystatin, and vincristine. In addition, ZawnVillines 2018 [145] has reported that carotenoids encapsulated in lipid bilayers can impair carotenoid stability. This, in turn, affects the properties of liposomes. This is because types of carotenoids and different concentrations could modulate the structure, hydrophobicity, and dynamics of the liposomal membrane [146]. In turn, the structural integrity of the liposome bilayers can protect the encapsulated compounds from degradation, thereby enhancing their physicochemical stability. In recent years, some liposomes have been manufactured for the simultaneous delivery of bioactive compounds. For example, pectin-coated phosphatidylcholine (PC) liposomes have been engineered to encapsulate two important lysozyme and nisin compounds [147].

**Figure 6:**
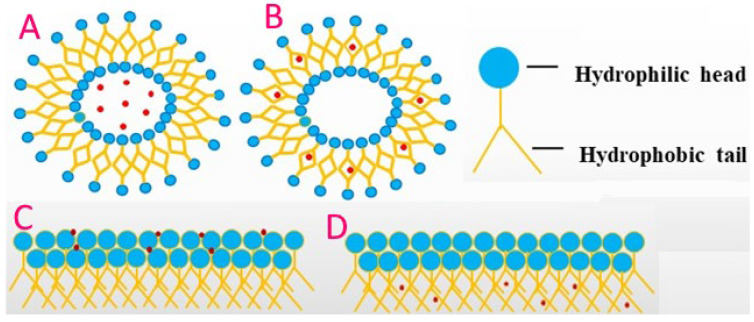
Schematic representation of bioactive compounds encapsulated in various nano-fabricated liposome systems. (**A**), bioactive compound entrapped in the interior of liposome region; (**B**), bioactive compound entrapped in the fatty acid region of liposome region; (**C**), bioactive compound entrapped in the hydrophilic head region of phospholipid bilayer; (**D**), bioactive compound entrapped in the hydrophobic head region of the phospholipid bilayer.

Furthermore, liposomes containing both the compounds lysozyme and nisin showed higher stability and antimicrobial activity compared to liposomes containing lysozyme, representing a promising co-delivery system for bioactive substances [148]. Water-soluble vitamin E derivative of PEG, d--tocopheryl polyethylene glycol 1000 succinate (TPGS). Such fabricated liposomes are more stable in biological environments and have proven to be a valuable, safe tool for incorporation into liposome membranes [149]. Some of the beneficial activities of liposomes include increased bioavailability and absorption rate compared to oral supplements; The micronized encapsulation protects against the harsh environment of the gastrointestinal tract and improves absorption [150]. It also has the potential to hold both hydrophilic and hydrophobic molecules. A list of Nutraceuticals encapsulated by liposomes is shown in Figure 6.

### 3.9. Solid Lipid Nanoparticles (SLNs) and Nanostructured Lipid Carrier (NLC)

(SLNs) and (NLC) are the other two important lipid-based nano delivery systems. SLNs are submicron emulsions established by homogenizing two immiscible phases in the presence of amphiphilic surfactant. These SLNs facilitate the maintenance of the liquid phase during the process. The lipid phase is melted at an elevated temperature to offer higher encapsulation efficiency, a slower degradation rate, and flexibility in the release profile [151]. The commonly used method for large-scale production of SLNs for food processing homogenization, the major problem associated with SLNs is the low encapsulation load. So that the manufacturing of nanostructured based lipid carrier as partially crystallized lipid nanocarriers provide an effective solution with enhanced physical stability, improved dispersibility, and higher encapsulation load as well as to move up a remarkable trap of hydrophilic and hydrophobic nutrients [152,153,154].

### 3.10. Nanogels

Nanogels are nanoparticles synthesized by combining hydrogel and a cross-linked chemically or physically with hydrophilic polymer chains [155]. It also signifies a biocompatible and biodegradable drug delivery system obtained from a nano-sized polymeric network; the swelling attains it under the action of a suitable solvent [156]. There are different ways to be administered these drug delivery systems. Nanogels can be expressed as they must be responsive to various stimuli involving magnetic field, temperature, and pH [157]. For example, the nanogels, which are made of dextran and ovalbumin, were produced by Maillard reaction and were effectively employed to improve curcumin bio-accessibility in the gastrointestinal tract, as was established in the form of an in vitro reproduced model [137].

## 4. Future Perspectives and Regulatory Outlook

The toxicological valuation of nano-fabricated material and their different delivery systems for bioactive compounds and nutraceuticals holds immense importance and vigilant assessment. The utilization of nano-fabricated materials may be allied with numerous safety reservations and necessitate implementing practices that perilously take safety and human health into consideration. The use of nano-fabricated materials in the food industry in food packaging is efficiently browbeaten with rapid growth but still coupled with end-user regulatory and safety issues that should be assessed and addressed critically [158] (Figure 6).

Health concerns, authoritarian policies, and safety must be well-thought-out during the production, processing, and wrapping of nano-based programmed food products. In terms of regulatory aspects of nanomaterials and nanostructure in the medical and food sector, there is no explicit legislation applied worldwide. Many countries still do not have specific authorization for nano-encapsulated products’ safety and risk assessments. The lack of reliability in the functional switching information between the countries is an outlined risk to mankind and the environment. It may perhaps minimize the promotion of novel and valuable products globally [159]. Due to the lack of specified regulations for food nanotechnology, European Union regulations and U.S. Food and Drug Administration has established guidelines regarding the use of nanotechnology in food and to get safety assessment approval before its use. In one of the articles on the nature of nanotechnology, Steffen Foss Hansen put forth a framework named ‘React Now,’ which stands for (Registration, Evaluation, Authorization, Categorization, and Tools to Evaluate Nanomaterials Opportunities and Weaknesses). All users of nano-fabricated materials should be evaluated according to the Nano-Risk-Cat-methodology [159]. Therefore, organizations and companies functioning with nano-fabricated materials have to carefully think about all the above-mentioned aspects to contest food safety concerns and ensure successful promotions of nano-fabricated programmed food products or nutraceuticals in the marketplace.

## 5. Conclusions

In this review, we focused on the biological functionality of bioactive compounds and nutraceuticals and their beneficial activity for maintaining a healthy lifestyle. Nutraceutical compounds derived from natural origin can treat several pathologies, inflammation, cancer, and cardiovascular diseases. In contrast, we reviewed the physicochemical instability of these significant compounds and declined bioavailability due to environmental conditions. Furthermore, we addressed the promising contribution of food technology throughout the sustained release/targeted delivery of bioactive compounds/nutraceuticals and about their defense from harsh environments through the use of various nanofabricated delivery systems. Various delivery systems such as carbohydrates, proteins, lipids (nano-emulsion, liposome-mediated delivery systems), and many other techniques have drawn scientific interest and allowed researchers to eradicate many of the limitations in using nutraceuticals. Food nanotechnology could be an alternative method to generate future trends in nutraceuticals and uphold bioactive compounds’ stability. However, certain regulatory aspects and safety issues need to be addressed by the European Union regulations and U.S. Food and Drug Administration to make the best use of nanofabricated materials as nanofabricated delivery systems to enhance the activity and retain the shelf-life of bioactive compounds and nutraceuticals to treat various disease and to protect or improve food constituents, flavor and taste for the betterment of mankind in maintaining a healthy standard of living.

## Figures and Tables

**Table 2:** Various nano-formulations systems of different bioactive compounds and their functional and physical properties.

Nano formulated Bioactive Compounds	Solubility	Stability	Nanofabricated Method/Source	Bioavailability/Release Kinetics	Main Findings	References
Rutin and Quercetin	Water-insoluble	Sensitive to light, oxidation, pH	PLGA	Release of quercetin ~64% after 3 days of injection, in vivo	Study of anticancer activity	[16]
Curcumin	Poor water solubility	Sensitive to oxygen, light	Curcumin Hydrogel beads (CHBs)	Cucumin release was 67% after 2 h, and 67% after 4 h	Mask bitterness, enhance solubility, and increase the bioavailability	[17]
Vitamin E	Water- insoluble	Sensitive to oxygen, light, pH	Oil –in water emulsion	Bioaccessibility of developed emulsions was in the range of 65–85%	Increased storage stability (Vitamin E fortified emulsions)	[18]
Vitamin E	Water -insoluble	Sensitive to oxygen, light, pH	Spiral dextrin inclusion complexes	95% of Vit E and 98% of soy isoflavone released after 80 min	Study of release kinetics of bioactive compound and Antioxidant capacity during the simulated gastrointestinal tract	[19]
Curcumin	Water-insoluble	Sensitive to light, heat, iron ion	Spi-Fuc polymer-core-shell nanoparticles	Release rates of curcumin were 96.25% and 82.69% after 4 h	Stability studies, delivering lipid soluble active ingredients	[20]
Curcumin and piperine	Water-insoluble	Sensitive to light, heat, iron ion	Nanoemulsion	Release of curcumin 40%, release of piperine 7.5% after 72 h	The activity of curcumin on HCT 116 Colorectal Cancer Model	[21]
Kaempferol glucoside	Water insoluble	Sensitive to oxidation, light, pH	Gold nanoparticles	-	Catalytic, antioxidant, and anticancer activities of gold nanoparticles	[22]
Berberine	Low water solubility	Sensitive to heat and pH	Liquid crystalline nanoparticle	80% of berberine released after 24 h	Anticancer activity in MCF7 human breast cancer cells	[23]
Carvacrol and linalool	Water insoluble	Sensitive to oxidation, light, pH	β-cyclodextrin-grafted chitosan	Carvacrol released—49% after 600 min, linalool released—71% after 460 min	sustainable biopesticide aiming pest control	[24]
Astaxanthin	Low water solubility	Sensitive to oxygen, light, heat, pH	Lupin protein-based Pickering emulsion	Astaxanthin powder exhibited 80% bioaccessibility	Usage as a food ingredientLupin protein-based particles	[25]

**Table 3:** List of probiotics and their applications.

Type	Micro-Organisms	Activity	Study	Reference
Probiotic	*Lactobacilli plantarum* C70	Anticancer effect	*Lactobacilli plantarum* C70 by releasing the exopolysaccharide, caused 73.1% and 88.1% cytotoxic properties against breast and colon cancers, respectively.	[117]
Probiotic	*Lactobacilli cocktail*	Anticancer effect	HT-29, a human colorectal carcinoma cell line, was controlled by *Lactobacilli cocktail* via the modulation of the Notch and Wnt/β-catenin signaling pathways	[118]
Probiotic	*L. rhamnosus*	Anticancer effect	The bioconversion of cranberry proanthocyanidins to *Lactobacillus rhamnosus* could result in the IC50 values of 20.1 and 47.8 µg/mL	[119]
Probiotic	*Eurotium cristatum*	Anti-obesity effect	The administration of *Eurotium cristatum* showed anti-obesity activity in mice fed a high-fat diet (HFD) through the modulation of gut microbiota	[120]

**Table 4:** List of prebiotics and their applications.

Type	Active Compounds	Activity	Study	Reference
Prebiotic	Chondroitin Sulfate Disaccharide	Anticancer effect	The growth of HT-29, human colon cancer cell line, was controlled by Chondroitin sulfate (CS)-Keel disaccharide (CSD) generated by chondroitin AC lyase, estimated at 80% antiproliferative activity.	[121]
Prebiotic	Blueberry anthocyanins	Antioxidant effect	The density and composition of intestinal microbiota in human models were increased by consumption of high purity blueberry anthocyanins through the increase in the modulatory and prebiotic activities.	[122]
Prebiotic	Short-chain fatty acids	Antiproliferative effects	The administration of short-chain fatty acids (SCFAs) prevented the expression of genes involved in the human colorectal cancer cell.	[123]
Prebiotic	Oligosaccharides	Antioxidant effect	The water-soluble oligosaccharide of EMOS-1a showed a 1420% proliferation level	[124]

**Table 5:** Nutraceutical encapsulation by the carbohydrate-based delivery system.

Nutraceuticals	Encapsulating Materials	Encapsulation Techniques	Target	Reference
Catechin	Azivash (*Corchorus olitorius* L.) gum-polyvinyl alcohol	Electrospinning process	Simulated gastric fluid and simulated intestinal fluid, EE%.	[131]
Hesperetin (HSP)	Nanofibers: Basil seed mucilage/polyvinylalcohol	Electrospinning	Characterization of nanofibers. Release models, EE%	[132]
Rutin	Quinoa and maize starch NPs	Ultrasonication	Characterization, EE%, simulated in vitro digestion.	[133]
Saffron Bioactive components	Nanoparticles: chitosan (CS) and gum arabic (GA)	Ionic gelation (IG)	Characterization NPs, EE%, Release of Saffron in acidic and natural media	[134]

## Data Availability

Data that support the findings are available within the manuscript.

## References

[1] Luo Y., Wang Q., Zhang Y. (2020). Biopolymer-Based Nanotechnology Approaches to Deliver Bioactive Compounds for Food Applications: A Perspective on the Past, Present, and Future. J. Agric. Food Chem..

[2] Daliu P., Santini A., Novellino E. (2019). From pharmaceuticals to nutraceuticals: Bridging disease prevention and management. Expert Rev. Clin. Pharmacol..

[3] Sarwal A., Rawat N., Singh G., Sinha V.R., Sharma S., Kumar D. (2018). Nanonutraceuticals in Central Nervous System Disorders. NanoNutraceuticals.

[4] Sasi S. (2017). Nutraceuticals—A review. Int. J. Ind. Biotechnol. Biomater..

[5] Deepika, Maurya P.K. (2022). Health Benefits of Quercetin in Age-Related Diseases. Molecules.

[6] Sagi S.S. (2021). Quercetin: A Potential Flavanol with Multiple Health Benefits. Arch. Food Sci. Nutr. Res..

[7] Yang L., Gao Y., Bajpai V.K., El-Kammar H.A., Simal-Gandara J., Cao H., Cheng K.W., Wang M., Arroo R.R., Zou L. (2021). Advance toward isolation, extraction, metabolism and health benefits of kaempferol, a major dietary flavonoid with future perspectives. Crit. Rev. Food Sci. Nutr..

[8] Guest P.C., Sahebkar A. (2021). Research in the Middle East into the Health Benefits of Curcumin. Studies on Biomarkers and New Targets in Aging Research in Iran.

[9] Xu X., Yi H., Wu J., Kuang T., Zhang J., Li Q., Du H., Xu T., Jiang G., Fan G. (2021). Therapeutic effect of berberine on metabolic diseases: Both pharmacological data and clinical evidence. Biomed. Pharmacother..

[10] Zhou L., Cai L., Ruan H., Zhang L., Wang J., Jiang H., Wu Y., Feng S., Chen J. (2021). Electrospun chitosan oligosaccharide/polycaprolactone nanofibers loaded with wound-healing compounds of Rutin and Quercetin as antibacterial dressings. Int. J. Biol. Macromol..

[11] Raza S.H.A., Naqvi S.R.Z., Abdelnour S.A., Schreurs N., Mohammedsaleh Z.M., Khan I., Shater A.F., El-Hack M.E.A., Khafaga A.F., Quan G. (2021). Beneficial effects and health benefits of Astaxanthin molecules on animal production: A review. Res. Vet. Sci..

[12] Zaaboul F., Liu Y. (2022). Vitamin E in foodstuff: Nutritional, analytical, and food technology aspects. Compr. Rev. Food Sci. Food Saf..

[13] Mangla B., Kohli K. (2018). Combination of natural agent with synthetic drug for the breast cancer therapy. Int. J. Drug Dev. Res..

[14] Majumdar M., Shivalkar S., Pal A., Verma M.L., Sahoo A.K., Roy D.N. (2019). Nanotechnology for enhanced bioactivity of bioactive compounds. Biotechnological Production of Bioactive Compounds.

[15] McClements D.J., Decker E., Weiss J. (2007). Emulsion-Based Delivery Systems for Lipophilic Bioactive Components. J. Food Sci..

[16] Ghanbari-Movahed M., Mondal A., Farzaei M.H., Bishayee A. (2022). Quercetin- and rutin-based nano-formulations for cancer treatment: A systematic review of improved efficacy and molecular mechanisms. Phytomedicine.

[17] Sharma M., Inbaraj B.S., Dikkala P.K., Sridhar K., Mude A.N., Narsaiah K. (2022). Preparation of Curcumin Hydrogel Beads for the Development of Functional *Kulfi*: A Tailoring Delivery System. Foods.

[18] Lv S., Zhang Y., Tan H., Zhang R., McClements D.J. (2019). Vitamin E Encapsulation within Oil-in-Water Emulsions: Impact of Emulsifier Type on Physicochemical Stability and Bioaccessibility. J. Agric. Food Chem..

[19] Wang P.-P., Luo Z.-G., Peng X.-C. (2018). Encapsulation of Vitamin E and Soy Isoflavone Using Spiral Dextrin: Comparative Structural Characterization, Release Kinetics, and Antioxidant Capacity during Simulated Gastrointestinal Tract. J. Agric. Food Chem..

[20] Fan L., Lu Y., Ouyang X.-K., Ling J. (2021). Development and characterization of soybean protein isolate and fucoidan nanoparticles for curcumin encapsulation. Int. J. Biol. Macromol..

[21] Bolat Z.B., Islek Z., Demir B.N., Yilmaz E.N., Sahin F., Ucisik M.H. (2020). Curcumin- and Piperine-Loaded Emulsomes as Combinational Treatment Approach Enhance the Anticancer Activity of Curcumin on HCT116 Colorectal Cancer Model. Front. Bioeng. Biotechnol..

[22] Oueslati M.H., Ben Tahar L., Harrath A.H. (2020). Catalytic, antioxidant and anticancer activities of gold nanoparticles synthesized by kaempferol glucoside from Lotus leguminosae. Arab. J. Chem..

[23] Loo Y.S., Madheswaran T., Rajendran R., Bose R.J. (2020). Encapsulation of berberine into liquid crystalline nanoparticles to enhance its solubility and anticancer activity in MCF7 human breast cancer cells. J. Drug Deliv. Sci. Technol..

[24] Campos E., Proença P.L.F., Oliveira J.L., Pereira A., Ribeiro L.N.D.M., Fernandes F.O., Gonçalves K.C., Polanczyk R.A., Pasquoto-Stigliani T., Lima R. (2018). Carvacrol and linalool co-loaded in β-cyclodextrin-grafted chitosan nanoparticles as sustainable biopesticide aiming pest control. Sci. Rep..

[25] Burgos-Díaz C., Opazo-Navarrete M., Soto-Añual M., Leal-Calderon F., Bustamante M. (2020). Food-grade Pickering emulsion as a novel astaxanthin encapsulation system for making powder-based products: Evaluation of astaxanthin stability during processing, storage, and its bioaccessibility. Food Res. Int..

[26] Aronson J.K. (2017). Defining ‘nutraceuticals’: Neither nutritious nor pharmaceutical. Br. J. Clin. Pharmacol..

[27] Prabu S.L., Suriyaprakash T., Dinesh K., Suresh K., Ragavendran T. (2012). Nutraceuticals: A review. Elixir Pharm..

[28] Crawford C., Boyd C., Paat C.F., Meissner K., Lentino C., Teo L., Berry K., Deuster P. (2019). Dietary Ingredients as an Alternative Approach for Mitigating Chronic Musculoskeletal Pain: Evidence-Based Recommendations for Practice and Research in the Military. Pain Med..

[29] Zanella M., Ciappellano S.G., Venturini M., Tedesco E., Manodori L., Benetti F. (2015). Nutraceuticals and nanotechnology. Diet. Ingred. Suppl..

[30] Subramani T., Ganapathyswamy H. (2020). An overview of liposomal nano-encapsulation techniques and its applications in food and nutraceutical. J. Food Sci. Technol..

[31] Singh A.R., Desu P.K., Nakkala R.K., Kondi V., Devi S., Alam M.S., Hamid H., Athawale R.B., Kesharwani P. (2022). Nanotechnology-based approaches applied to nutraceuticals. Drug Deliv. Transl. Res..

[32] Sahani S., Sharma Y.C. (2021). Advancements in applications of nanotechnology in global food industry. Food Chem..

[33] Manocha S., Dhiman S., Grewal A.S., Guarve K. (2022). Nanotechnology: An approach to overcome bioavailability challenges of nutraceuticals. J. Drug Deliv. Sci. Technol..

[34] Bronzwaer S., Kass G., Robinson T., Tarazona J., Verhagen H., Verloo D., Vrbos D., Hugas M. (2019). Food Safety Regulatory Research Needs 2030. EFSA J..

[35] Whitman M. (2001). Understanding the perceived need for complementary and alternative nutraceuticals: Lifestyle issues. Clin. J. Oncol Nurs..

[36] Ghani U., Naeem M., Rafeeq H., Imtiaz U., Amjad A., Ullah S., Rehman A., Qasim F. (2019). A Novel Approach towards Nutraceuticals and Biomedical Applications. Sch. Int. J. Biochem..

[37] Palmer M.E., Haller C., McKinney P.E., Klein-Schwartz W., Tschirgi A., Smolinske S.C., Woolf A., Sprague B.M., Ko R., Everson G. (2003). Adverse events associated with dietary supplements: An observational study. Lancet.

[38] Dable-Tupas G., Otero M.C.B., Bernolo L. (2020). Functional foods and health benefits. Functional Foods and Nutraceuticals.

[39] Crowe K.M., Francis C. (2013). Position of the Academy of Nutrition and Dietetics: Functional Foods. J. Acad. Nutr. Diet..

[40] Dwyer J.T., Coates P.M., Smith M.J. (2018). Dietary Supplements: Regulatory Challenges and Research Resources. Nutrients.

[41] Rawson E.S., Miles M.P., Larson-Meyer D.E. (2018). Dietary supplements for health, adaptation, and recovery in athletes. Int. J. Sport Nutr. Exerc. Metab..

[42] Psota T.L., Gebauer S.K., Kris E.P. (2006). Dietary omega-3 fatty acid intake and cardiovascular risk. Am. J. Cardiol..

[43] Chandra S., Saklani S., Kumar P., Kim B., Coutinho H.D.M. (2022). Nutraceuticals: Pharmacologically Active Potent Dietary Supplements. BioMed Res. Int..

[44] Abbadi A., Domergue F. (2004). Biosynthesis of very long- chain polyunsaturated fatty acids in transgenic oilseeds: Constraints on their accumulation. Plant Cell.

[45] Wu G., Truksa M., Datla N. (2005). Stepwise engineering to produce high yields of very long-chain polyunsaturated fatty acids in plants. Nat. Biotechnol..

[46] Das L., Bhaumik E., Raychaudhuri U., Chakraborty R. (2012). Role of nutraceuticals in human health. J. Food Sci. Technol..

[47] Siciliano R., Reale A., Mazzeo M., Morandi S., Silvetti T., Brasca M. (2021). Paraprobiotics: A New Perspective for Functional Foods and Nutraceuticals. Nutrients.

[48] Adefegha A. (2018). Functional Foods and Nutraceuticals as Dietary Intervention in Chronic Diseases; Novel Perspectives for Health Promotion and Disease Prevention. J. Diet. Suppl..

[49] Gruss S.M., Nhim K., Gregg E., Bell M., Luman E., Albright A. (2019). Public Health Approaches to Type 2 Diabetes Prevention: The US National Diabetes Prevention Program and Beyond. Curr. Diabetes Rep..

[50] De Felice S. FIM rationale and proposed guidelines for the nutraceutical research & education act–NREA. Proceedings of the FIM’s 10th Nutraceutical Conference.

[51] Rajasekaran A., Sivagnanam G., Xavier R. (2008). Nutraceuticals as therapeutic agents, a review research. Res. J. Pharm. Technol..

[52] Ozdal T., Tomas M., Toydemir G., Kamiloglu S., Capanoglu E. (2021). Introduction to nutraceuticals, medicinal foods, and herbs. Aromatic Herbs in Food.

[53] Jagannathan R., Patel S.A., Ali M.K., Narayan K.M.V. (2019). Global Updates on Cardiovascular Disease Mortality Trends and Attribution of Traditional Risk Factors. Curr. Diabetes Rep..

[54] Inoue M., Sumii Y., Shibata N. (2020). Contribution of Organofluorine Compounds to Pharmaceuticals. ACS Omega.

[55] Gonçalves R.F.S., Martins J.T., Duarte C.M.M., Vicente A.A., Pinheiro A.C. (2018). Advances in nutraceutical delivery systems: From formulation design for bioavailability enhancement to efficacy and safety evaluation. Trends Food Sci. Technol..

[56] Gopi S., Balakrishnan P. (2022). Handbook of Nutraceuticals and Natural Products.

[57] Shahgholian N. (2002). Introduction to Nutraceuticals and Natural Products. Handbook of Nutraceuticals and Natural Products: Biological, Medicinal, and Nutritional Properties and Applications.

[58] Durazzo A., Lucarini M., Santini A. (2020). Nutraceuticals in Human Health. Foods.

[59] Natarajan T.D., Ramasamy J.R., Palanisamy K. (2019). Nutraceutical potentials of synergic foods: A systematic review. J. Ethn. Foods.

[60] Ghaffari S., Roshanravan N. (2020). The role of nutraceuticals in prevention and treatment of hypertension: An updated review of the literature. Food Res. Int..

[61] Roshanravan N., Mohammadi H. (2019). The Role of Nutraceuticals in Gestational Diabetes Mellitus. Nutraceuticals for Prenatal, Maternal and Offspring’s Nutritional Health.

[62] Allahveran A., Farokhzad A., Asghari M., Sarkhosh A. (2018). Foliar application of ascorbic and citric acids enhanced ‘Red Spur’apple fruit quality, bioactive compounds and antioxidant activity. Physiol. Mol. Biol. Plants.

[63] Langer R., Peppas N.A. (2003). Advances in biomaterials, drug delivery, and bionanotechnology. AIChE J..

[64] Estevinho L.M. (2018). Special issue “Nutraceuticals in human health and disease”. Int. J. Mol. Sci..

[65] Ting Y., Jiang Y., Ho C.-T., Huang Q. (2014). Common delivery systems for enhancing in vivo bioavailability and biological efficacy of nutraceuticals. J. Funct. Foods.

[66] Meléndez-Martínez A.J., Böhm V., Borge G.I.A., Cano M.P., Fikselová M., Gruskiene R., Lavelli V., Loizzo M.R., Mandić A.I., Brahm P.M. (2021). Carotenoids: Considerations for Their Use in Functional Foods, Nutraceuticals, Nutricosmetics, Supplements, Botanicals, and Novel Foods in the Context of Sustainability, Circular Economy, and Climate Change. Annu. Rev. Food Sci. Technol..

[67] Leonard N.B. (2000). Stability testing of nutraceuticals and functional foods. Handbook of Nutraceuticals and Functional Foods.

[68] Zheng B., McClements D.J. (2020). Formulation of More Efficacious Curcumin Delivery Systems Using Colloid Science: Enhanced Solubility, Stability, and Bioavailability. Molecules.

[69] Jones D., Caballero S., Davidov-Pardo G. (2019). Chapter Six—Bioavailability of nanotechnology-based bioactives and nutraceuticals. Advances in Food and Nutrition Research.

[70] Punia S., Sandhu K.S., Kaur M., Siroha A.K. (2019). Nanotechnology: A successful approach to improve nutraceutical bioavailability. Nanobiotechnology in Bioformulations.

[71] Helal N.A., Eassa H.A., Amer A.M., Eltokhy M.A., Edafiogho I., Nounou M.I. (2019). Nutraceuticals’ novel formulations: The good, the bad, the unknown and patents involved. Recent Pat. Drug Deliv. Formul..

[72] Prasad R., Kumar V., Kumar M., Choudhary D.K. (2019). Nanobiotechnology in Bioformulations.

[73] Huang Q., Yu H., Ru Q. (2010). Bioavailability and Delivery of Nutraceuticals Using Nanotechnology. J. Food Sci..

[74] Pandey M., Verma R.K., Saraf S.A. (2010). Nutraceuticals: New era of medicine and health. Asian J. Pharm. Clin. Res..

[75] Rachmale M., Rajput N., Jadav T., Sahu A.K., Tekade R.K., Sengupta P. (2022). Implication of metabolomics and transporter modulation based strategies to minimize multidrug resistance and enhance site-specific bioavailability: A needful consideration toward modern anticancer drug discovery. Drug Metab. Rev..

[76] Balkrishna A., Sakat S.S., Joshi K., Joshi K., Sharma V., Ranjan R., Bhattacharya K., Varshney A. (2019). Cytokines driven anti-Inflammatory and anti-psoriasis like efficacies of nutraceutical sea buckthorn (hippophaerhamnoides) oil. Front. Pharm..

[77] Younis S.A., Kim K.-H., Shaheen S.M., Antoniadis V., Tsang Y.F., Rinklebe J., Deep A., Brown R.J. (2021). Advancements of nanotechnologies in crop promotion and soil fertility: Benefits, life cycle assessment, and legislation policies. Renew. Sustain. Energy Rev..

[78] Bortolotti M., Mercatelli D., Polito L. (2019). Momordica charantia, a Nutraceutical Approach for Inflammatory Related Diseases. Front. Pharmacol..

[79] Gonçalves A., Estevinho B.N., Rocha F. (2021). Methodologies for simulation of gastrointestinal digestion of different controlled delivery systems and further uptake of encapsulated bioactive compounds. Trends Food Sci. Technol..

[80] DeRosa G., Limas C.P., Macías P.C., Estrella A., Maffioli P. (2014). Dietary and nutraceutical approach to type 2 diabetes. Arch. Med Sci..

[81] Sachdeva V., Roy A., Bharadvaja N. (2020). Current Prospects of Nutraceuticals: A Review. Curr. Pharm. Biotechnol..

[82] Castrogiovanni P., Trovato F.M., Loreto C., Nsir H., Szychlinska M.A., Musumeci G. (2016). Nutraceutical Supplements in the Management and Prevention of Osteoarthritis. Int. J. Mol. Sci..

[83] Pashte V.V., Pashte S.V., Said P.P. (2020). Nutraceutical properties of natural honey to fight health issues: A comprehensive review. J. Pharmacogn. Phytochem..

[84] Abbas M., Shah I., Nawaz M.A., Pervez S., Hussain Y., Niaz K., Khan F. (2021). Honey Against Cancer. Nutraceuticals and Cancer Signaling.

[85] Ajibola A., Chamunorwa J.P., Erlwanger K.H. (2012). Nutraceutical values of natural honey and its contribution to human health and wealth. Nutr. Metab..

[86] Cianciosi D., Forbes-Hernández T.Y., Ansary J., Gil E., Amici A., Bompadre S., Simal-Gandara J., Giampieri F., Battino M. (2020). Phenolic compounds from Mediterranean foods as nutraceutical tools for the prevention of cancer: The effect of honey polyphenols on colorectal cancer stem-like cells from spheroids. Food Chem..

[87] Cicero A.F.G., Fogacci F., Bove M., Giovannini M., Borghi C. (2019). Three-arm, placebo-controlled, randomized clinical trial evaluating the metabolic effect of a combined nutraceutical containing a bergamot standardized flavonoid extract in dyslipidemic overweight subjects. Phytother. Res..

[88] Martínez-Puc J.F., Cetzal-Ix W., Basu S.K., Enríquez-Nolasco J.R., Magaña-Magaña M.A. Nutraceutical and medicinal properties of native stingless bees honey and their contribution to human health. In Functional Foods and Nutraceuticals in Metabolic and Non-Communicable Diseases, Academic Press: Cambridge, MA, USA, 2022; pp. 481–489.

[89] Poli A., Barbagallo C.M., Cicero A.F., Corsini A., Manzato E., Trimarco B., Bernini F., Visioli F., Bianchi A., Canzone G. (2018). Nutraceuticals and functional foods for the control of plasma cholesterol levels. An intersociety position paper. Pharmacol. Res..

[90] Cicero A.F.G., Fogacci F., Stoian A.P., Vrablik M., Al Rasadi K., Banach M., Toth P.P., Rizzo M. (2021). Nutraceuticals in the Management of Dyslipidemia: Which, When, and for Whom? Could Nutraceuticals Help Low-Risk Individuals with Non-optimal Lipid Levels?. Curr. Atheroscler. Rep..

[91] Chen Y., Pan H., Hao S., Pan D., Wang G., Yu W. (2021). Evaluation of phenolic composition and antioxidant properties of different varieties of Chinese citrus. Food Chem..

[92] Alcaide-Hidalgo J.M., Romero M., Duarte J., López-Huertas E. (2020). Antihypertensive Effects of Virgin Olive Oil (Unfiltered) Low Molecular Weight Peptides with ACE Inhibitory Activity in Spontaneously Hypertensive Rats. Nutrients.

[93] Akbari B., Baghaei-Yazdi N., Bahmaie M., Abhari F.M. (2022). The role of plant-derived natural antioxidants in reduction of oxidative stress. BioFactors.

[94] Sahebkar A., Serban M.-C., Gluba-Brzózka A., Mikhailidis D.P., Cicero A.F., Rysz J., Banach M. (2016). Lipid-modifying effects of nutraceuticals: An evidence-based approach. Nutrition.

[95] Bianconi V., Mannarino M.R., Sahebkar A., Cosentino T., Pirro M. (2018). Cholesterol-lowering nutraceuticals affecting vascular function and cardiovascular disease risk. Curr. Cardiol. Rep..

[96] Paolino D., Mancuso A., Cristiano M., Froiio F., Lammari N., Celia C., Fresta M. (2021). Nanonutraceuticals: The New Frontier of Supplementary Food. Nanomaterials.

[97] Liu X., Raghuvanshi R., Ceylan F.D., Bolling B.W. (2020). Quercetin and Its Metabolites Inhibit Recombinant Human Angiotensin-Converting Enzyme 2 (ACE2) Activity. J. Agric. Food Chem..

[98] Cimaglia P., Sega F.V.D., Vitali F., Lodolini V., Bernucci D., Passarini G., Fortini F., Marracino L., Aquila G., Rizzo P. (2019). Effectiveness of a Novel Nutraceutical Compound Containing Red Yeast Rice, Polymethoxyflavones and Antioxidants in the Modulation of Cholesterol Levels in Subjects with Hypercholesterolemia and Low-Moderate Cardiovascular Risk: The NIRVANA Study. Front. Physiol..

[99] Barreca D., Laganà G., Leuzzi U., Smeriglio A., Trombetta D., Bellocco E. (2016). Evaluation of the nutraceutical, antioxidant and cytoprotective properties of ripe pistachio (*Pistacia vera* L., variety Bronte) hulls. Food Chem..

[100] Mandalari G., Barreca D., Gervasi T., Roussell M.A., Klein B., Feeney M.J., Carughi A. (2021). Pistachio Nuts (*Pistacia vera* L.): Production, Nutrients, Bioactives and Novel Health Effects. Plants.

[101] Kumar K., Yadav A.N., Kumar V., Vyas P., Dhaliwal H.S. (2017). Food waste: A potential bioresource for extraction of NutraceuticalsAnd bioactive compounds. Bioresour. Bioprocess..

[102] Tenore G.C., Caruso D., D’Avino M., Buonomo G., Caruso G., Ciampaglia R., Schiano E., Maisto M., Annunziata G., Novellino E. (2020). A Pilot Screening of Agro-Food Waste Products as Sources of Nutraceutical Formulations to Improve Simulated Postprandial Glycaemia and Insulinaemia in Healthy Subjects. Nutrients.

[103] Varzakas T., Zakynthinos G., Verpoort F. (2016). Plant Food Residues as a Source of Nutraceuticals and Functional Foods. Foods.

[104] Enrico C. (2019). Nanotechnology-Based Drug Delivery of Natural Compounds and Phytochemicals for the Treatment of Cancer and Other Diseases. Studies in Natural Products Chemistry.

[105] Khorasani S., Danaei M., Mozafari M. (2018). Nanoliposome technology for the food and nutraceutical industries. Trends Food Sci. Technol..

[106] Salehi B., Venditti A., Sharifi-Rad M., Kręgiel D., Sharifi-Rad J., Durazzo A., Martins N. (2019). The therapeutic potential of apigenin. Int. J. Mol. Sci..

[107] Tahir A., Ahmad R.S., Imran M., Ahmad M.H., Khan M.K., Muhammad N., Nisa M.U., Nadeem M.T., Yasmin A., Tahir H.S. (2021). Recent approaches for utilization of food components as nano-encapsulation: A review. Int. J. Food Prop..

[108] Patra J.K., Das G., Fraceto L.F., Campos E.V.R., del Pilar Rodriguez-Torres M., Acosta-Torres L.S., Diaz-Torres L.A., Grillo R., Swamy M.K., Sharma S. (2018). Nano based drug delivery systems: Recent developments and future prospects. J. Nanobiotechnol..

[109] Martínez-Ballesta M., Gil-Izquierdo Á., García-Viguera C., Domínguez-Perles R. (2018). Nanoparticles and controlled delivery for bioactive compounds: Outlining challenges for new “smart-foods” for health. Foods.

[110] Persano F., Gigli G., Leporatti S. (2021). Lipid-polymer hybrid nanoparticles in cancer therapy: Current overview and future directions. Nano Express.

[111] Mitchell M.J., Billingsley M.M., Haley R.M., Wechsler M.E., Peppas N.A., Langer R. (2021). Engineering precision nanoparticles for drug delivery. Nat. Rev. Drug Discov..

[112] Sosa-Hernández J.E., Villalba-Rodríguez A.M., Romero-Castillo K.D., Zavala-Yoe R., Bilal M., Ramirez-Mendoza R.A., Parra-Saldivar R., Iqbal H.M. (2020). Poly-3-hydroxybutyrate-based constructs with novel characteristics for drug delivery and tissue engineering applications—A review. Polym. Eng. Sci..

[113] Sonawane P.G., Doshi Y.S., Shah M.U., Bajaj M., Kevadia V. (2017). Probiotics: The Nano Soldiers for Periodontium. J. PEARLDENT.

[114] Wang X., Cheng F., Wang X., Feng T., Xia S., Zhang X. (2021). Chitosan decoration improves the rapid and long-term antibacterial activities of cinnamaldehyde-loaded liposomes. Int. J. Biol. Macromol..

[115] Hills S., Walker M., Barry A.E. (2019). Sport as a vehicle for health promotion: A shared value example of corporate social responsibility. Sport Manag. Rev..

[116] Khangwal I., Yadav M., Mandeep, Shukla P. (2020). Probiotics and Prebiotics: Techniques Used and Its Relevance. Microbial Enzymes and Biotechniques.

[117] Ayyash M., Abu-Jdayil B., Itsaranuwat P., Galiwango E., Tamiello-Rosa C., Abdullah H., Esposito G., Hunashal Y., Obaid R.S., Hamed F. (2020). Characterization, bioactivities, and rheological properties of exopolysaccharide produced by novel probiotic Lactobacillus plantarum C70 isolated from camel milk. Int. J. Biol. Macromol..

[118] Ghanavati R., Asadollahi P., Shapourabadi M.B., Razavi S., Talebi M., Rohani M. (2020). Inhibitory effects of Lactobacilli cocktail on HT-29 colon carcinoma cells growth and modulation of the Notch and Wnt/β-catenin signaling pathways. Microb. Pathog..

[119] Rupasinghe H.P.V., Parmar I., Neir S.V. (2019). Biotransformation of Cranberry Proanthocyanidins to Probiotic Metabolites by *Lactobacillus rhamnosus* Enhances Their Anticancer Activity in HepG2 Cells In Vitro. Oxidative Med. Cell. Longev..

[120] Kang D., Su M., Duan Y., Huang Y. (2019). *Eurotium cristatum*, a potential probiotic fungus from Fuzhuan brick tea, alleviated obesity in mice by modulating gut microbiota. Food Funct..

[121] Rani A., Baruah R., Goyal A. (2019). Prebiotic Chondroitin Sulfate Disaccharide Isolated from Chicken Keel Bone Exhibiting Anticancer Potential Against Human Colon Cancer Cells. Nutr. Cancer.

[122] Zhou L., Xie M., Yang F., Liu J. (2020). Antioxidant activity of high purity blueberry anthocyanins and the effects on human intestinal microbiota. LWT.

[123] Ohara T., Mori T. (2019). Antiproliferative Effects of Short-chain Fatty Acids on Human Colorectal Cancer Cells *via* Gene Expression Inhibition. Anticancer Res..

[124] Li E., Yang S., Zou Y., Cheng W., Li B., Hu T., Li Q., Wang W., Liao S., Pang D. (2019). Purification, Characterization, Prebiotic Preparations and Antioxidant Activity of Oligosaccharides from Mulberries. Molecules.

[125] Davani-Davari D., Negahdaripour M., Karimzadeh I., Seifan M., Mohkam M., Masoumi S.J., Ghasemi Y. (2019). Prebiotics: Definition, types, sources, mechanisms, and clinical applications. Foods.

[126] Pandey K., Naik S., Vakil B. (2015). Probiotics, prebiotics and synbiotics—a review. J. Food Sci. Technol..

[127] Nuruzzaman M., Rahman M.M., Liu Y., Naidu R. (2016). Nanoencapsulation, Nano-guard for Pesticides: A New Window for Safe Application. J. Agric. Food Chem..

[128] Xiao J., Cao Y., Huang Q. (2017). Edible Nanoencapsulation Vehicles for Oral Delivery of Phytochemicals: A Perspective Paper. J. Agric. Food Chem..

[129] Fathi M., Martín A., McClements D.J. (2014). Nanoencapsulation of food ingredients using carbohydrate based delivery systems. Trends Food Sci. Technol..

[130] Samaranayaka A.G., Li-Chan E.C. (2011). Food-derived peptidic antioxidants: A review of their production, assessment, and potential applications. J. Funct. Foods.

[131] Hoseyni S.Z., Jafari S.M., Tabarestani H.S., Ghorbani M., Assadpour E., Sabaghi M. (2021). Release of catechin from Azivash gum-polyvinyl alcohol electrospun nanofibers in simulated food and digestion media. Food Hydrocoll..

[132] Kurd F., Fathi M., Shekarchizadeh H. (2019). Nanoencapsulation of hesperetin using basil seed mucilage nanofibers: Characterization and release modeling. Food Biosci..

[133] Remanan M.K., Zhu F. (2021). Encapsulation of rutin using quinoa and maize starch nanoparticles. Food Chem..

[134] Rajabi H., Jafari S.M., Rajabzadeh G., Sarfarazi M., Sedaghati S. (2020). Chitosan-gum Arabic complex nanocarriers for encapsulation of saffron bioactive components. Colloids Surf. A Physicochem. Eng. Asp..

[135] Fathi M., Donsi F., McClements D.J. (2018). Protein-Based Delivery Systems for the Nanoencapsulation of Food Ingredients. Compr. Rev. Food Sci. Food Saf..

[136] Ahmad U., Ali A., Khan M.M., Siddiqui M.A., Akhtar J., Ahmad F.J. (2019). Nanotechnology-Based Strategies for Nutraceuticals: A Review of Current Research Development. Nanosci. Technol. Int. J..

[137] Zhang R., Han Y., Xie W., Liu F., Chen S. (2022). Advances in Protein-Based Nanocarriers of Bioactive Compounds: From Microscopic Molecular Principles to Macroscopical Structural and Functional Attributes. J. Agric. Food Chem..

[138] Mozafari M.R., Flanagan J., Matia-Merino L., Awati A., Omri A., Suntres Z.E., Singh H. (2006). Recent trends in the lipid-based nanoencapsulation of antioxidants and their role in foods. J. Sci. Food Agric..

[139] Akhavan S., Assadpour E., Katouzian I., Jafari S.M. (2018). Lipid Nano Scale Cargos for The Protection and Delivery of Food Bioactive Ingredients and Nutraceuticals. Trends Food Sci. Technol..

[140] Livney Y.D. (2015). Nanostructured delivery systems in food: Latest developments and potential future directions. Curr. Opin. Food Sci..

[141] Assadpour E., Jafari S.M., Jafari S. (2019). An overview of specialized equipment for nanoencapsulation of food ingredients. Nanoencapsulation of Food Ingredients by Specialized Equipment.

[142] Rashidi L. (2021). Different nano-delivery systems for delivery of nutraceuticals. Food Biosci..

[143] Ajeeshkumar K.K., Aneesh P.A., Raju N., Suseela M., Ravishankar C.N., Benjakul S. (2021). Advancements in liposome technology: Preparation techniques and applications in food, functional foods, and bioactive delivery: A review. Compr. Rev. Food Sci. Food Saf..

[144] Rostamabadi H., Falsafi S.R., Jafari S.M. (2019). Nanoencapsulation of carotenoids within lipid-based nanocarriers. J. Control. Release.

[145] Xia S., Tan C., Zhang Y., Abbas S., Feng B., Zhang X., Qin F. (2015). Modulating effect of lipid bilayer–carotenoid interactions on the property of liposome encapsulation. Colloids Surf. B Biointerfaces.

[146] Sercombe L., Veerati T., Moheimani F., Wu S.Y., Sood A.K., Hua S. (2015). Advances and Challenges of Liposome Assisted Drug Delivery. Front. Pharmacol..

[147] Lopes N.A., Pinilla C.M.B., Brandelli A. (2019). Antimicrobial activity of lysozyme-nisin co-encapsulated in liposomes coated with polysaccharides. Food Hydrocoll..

[148] Zhou W., Cheng C., Ma L., Zou L., Liu W., Li R., Cao Y., Liu Y., Ruan R., Li J. (2021). The Formation of Chitosan-Coated Rhamnolipid Liposomes Containing Curcumin: Stability and In Vitro Digestion. Molecules.

[149] Dalmoro A., Bochicchio S., Lamberti G., Bertoncin P., Janssens B., Barba A.A. (2019). Micronutrients encapsulation in enhanced nanoliposomal carriers by a novel preparative technology. RSC Adv..

[150] Peng Y., Meng Q., Zhou J., Chen B., Xi J., Long P., Zhang L., Hou R. (2019). Nanoemulsion delivery system of tea polyphenols enhanced the bioavailability of catechins in rats. Food Chem..

[151] Katouzian I., Esfanjani A.F., Jafari S.M., Akhavan S. (2017). Formulation and application of a new generation of lipid nano-carriers for the food bioactive ingredients. Trends Food Sci. Technol..

[152] Fathi M., Mozafari M.R., Mohebbi M. (2012). Nanoencapsulation of food ingredients using lipid based delivery systems. Trends Food Sci. Technol..

[153] Dhiman N., Awasthi R., Sharma B., Kharkwal H., Kulkarni G.T. (2021). Lipid nanoparticles as carriers for bioactive delivery. Front. Chem..

[154] Zhang Z., Li X., Sang S., McClements D.J., Chen L., Long J., Jiao A., Wang J., Jin Z., Qiu C. (2022). A review of nanostructured delivery systems for the encapsulation, protection, and delivery of silymarin: An emerging nutraceutical. Food Res. Int..

[155] Peres L.B., dos Anjos R.S., Tappertzhofen L.C., Feuser P.E., de Araújo P.H., Landfester K., Muñoz-Espí R. (2018). pH-responsive physically and chemically cross-linked glutamic-acid-based hydrogels and nanogels. Eur. Polym. J..

[156] Sahu P., Kashaw S.K., Sau S., Kushwah V., Jain S., Agrawal R.K., Iyer A.K. (2019). pH Responsive 5-Fluorouracil Loaded Biocompatible Nanogels for Topical Chemotherapy of Aggressive Melanoma. Colloids Surf. B Biointerfaces.

[157] Wang J., Xu W., Zhang N., Yang C., Xu H., Wang Z., Li B., Ding J., Chen X. (2021). X-ray-responsive polypeptide nanogel for concurrent chemoradiotherapy. J. Control. Release.

[158] Mullen E., Morris M. (2021). Green Nanofabrication Opportunities in the Semiconductor Industry: A Life Cycle Perspective. Nanomaterials.

[159] Bazana M.T., Codevilla C.F., de Menezes C.R. (2019). Nanoencapsulation of bioactive compounds: Challenges and perspectives. Curr. Opin. Food Sci..

